# Radiation-induced late dysphagia after intensity-modulated radiotherapy in nasopharyngeal carcinoma patients: a dose-volume effect analysis

**DOI:** 10.1038/s41598-018-34803-y

**Published:** 2018-11-06

**Authors:** Li Jiang, Chenhui Huang, Yixiu Gan, Tong Wu, Xiaobi Tang, Yiru Wang, Rensheng Wang, Yong Zhang

**Affiliations:** 1 Department of Radiation Oncology, The First Affiliated Hospital of Guangxi Medical University, Radiation Oncology Clinical Medical Research Center of Guangxi, Nanning, Guangxi China; 2 Guangxi Clinical Research Center for Digital Medicine and 3D Printing, Guigang City People’s Hospital, Guigang, Guangxi China

## Abstract

Dysphagia is a side effect of nasopharyngeal carcinoma chemo-radiotherapy (CRT) which greatly influences the quality of life of the patients. We analyzed late dysphagia in 134 patients with nasopharyngeal cancer undergoing radical radiotherapy (RT), and correlated these findings with dose–volume histogram (DVH) parameters of the swallowing organs at risk (SWOARs). DVH parameters of SWOARs were correlated with late dysphagia, and with RTOG/EORTC scale score and the M. D. Anderson dysphagia inventory (MDADI) score. The mean dose (D_mean_) to the superior and inferior constrictor muscles (SCM and ICM) and age were associated with grade 2 late dysphagia. Receiver operating characteristic (ROC) curves showed that the threshold values for grade 2 late dysphagia were: D_mean_ to SCM ≥ 67 Gy, partial volume receiving specified dose of 60 Gy (V_60_) of SCM ≥ 95%, D_mean_ to ICM ≥ 47 Gy, and V_50_ of ICM ≥ 23%. The areas under the ROC curve were 0.681 (p = 0.02), 0.677 (p = 0.002), 0.71 (p < 0.001) and 0.726 (p < 0.001) respectively. Our study demonstrates a significant relationship between late dysphagia and the radiation doses delivered to the SCM and ICM. Our findings suggest that physicians should be cautious in reducing the RT dose to SWOARs in order to avoid severe dysphagia. Further prospective trials are necessary to recommend this as part of routine clinical practice.

## Introduction

Nasopharyngeal carcinoma (NPC) is a common malignancy in Southern China, especially in Guangdong and Guangxi provinces^1,2^. Radiotherapy is the primary treatment strategy for non-disseminated NPC due to its unique anatomical position and radio-sensitivity. In recent years, intensity-modulated radiation therapy (IMRT), which is characterized by high conformity and the benefit of sparing the organs at risk (OARs), has been used for NPC treatment. Compared to conventional radiotherapy, IMRT reduces the incidence of several complications such as oral mucositis, xerostomia and temporal lobe injury, and improves patients’ quality of life (QOL)^3^. Late dysphagia has however emerged as a common side effect of NPC radiotherapy^4,5^.

Normal swallowing is a complex process involving several muscles and cranial nerves. Late dysphagia is associated with swallowing-related structures, tumor, and treatment^6,7^. However, the IMRT dose tolerances of the swallowing-related structures are poorly characterized. Furthermore, much of the existing data is based on the experiences of the 2D-CRT era, and concrete clinical evidence regarding IMRT is lacking^8^. Therefore, it is necessary to further study the radio-tolerance of SWOARs in NPC patients receiving IMRT.

We retrospectively reviewed the clinical and dosimetric data of a cohort of NPC patients who developed late dysphagia after IMRT, and performed dose-volume outcome analysis to determine the effect of different doses on swallowing-related structures. The aim was to evaluate the potential relationship between the planned dose–volume parameters and the observed incidence of late dysphagia in these patients. After a re-contouring of the SWOARs according to recently published guidelines, we determined the dose tolerated by the SWOARs that achieved the highest uncomplicated tumor control.

## Materials and Methods

### Patient population

A total of 158 patients with NPC who received IMRT at the First Affiliated Hospital of Guangxi Medical University from March 2013 to April 2014 were initially enrolled. The inclusion criteria were 1) treatment with curative IMRT at a dose delivered to the gross tumor volume (PGTVnx) of at least 66 Gy either alone or in combination with concomitant chemotherapy, 2) the availability of treatment plan record with DVH parameters, and 3) willingness to complete the stipulated questionnaires. The exclusion criteria were: 1) persistent/recurrent tumor, 2) distant metastasis, 3) previous radiotherapy for another head and neck tumor or with palliative intent, 4) Any RTOG/EORTC grade swallowing dysfunction before treatment and tumor invasion in oropharynx and hypopharynx, and 5) hoarseness, nasal regurgitation, lingual deviation and atrophy, coughing while drinking, unclear enunciation etc. The newly diagnosed NPC patients who suffered from dysphagia before treatment were still excluded so as to ensure that the observed dysphagia was only induced by radiation-related SWOARs dysfunction but not by lower cranial neuropathy. Based on these criteria, 24 patients were excluded: 3 were lost during the follow-up, 15 underwent a second treatment (re-irradiation and/or chemotherapy) due to either distant metastasis (n = 9) or loco-regional relapse (n = 6), and 6 were excluded because they had swallowing dysfunction before treatment. The remaining 134 patients received the questionnaires and provided informed consent. All methods were in accordance with the relevant guidelines and regulations, and were approved by the Ethical Review Committee of the First Affiliated Hospital of Guangxi Medical University. All clinical information of the participants is available and can be accessed.

### Outline of swallowing structures

**Figure 1:**
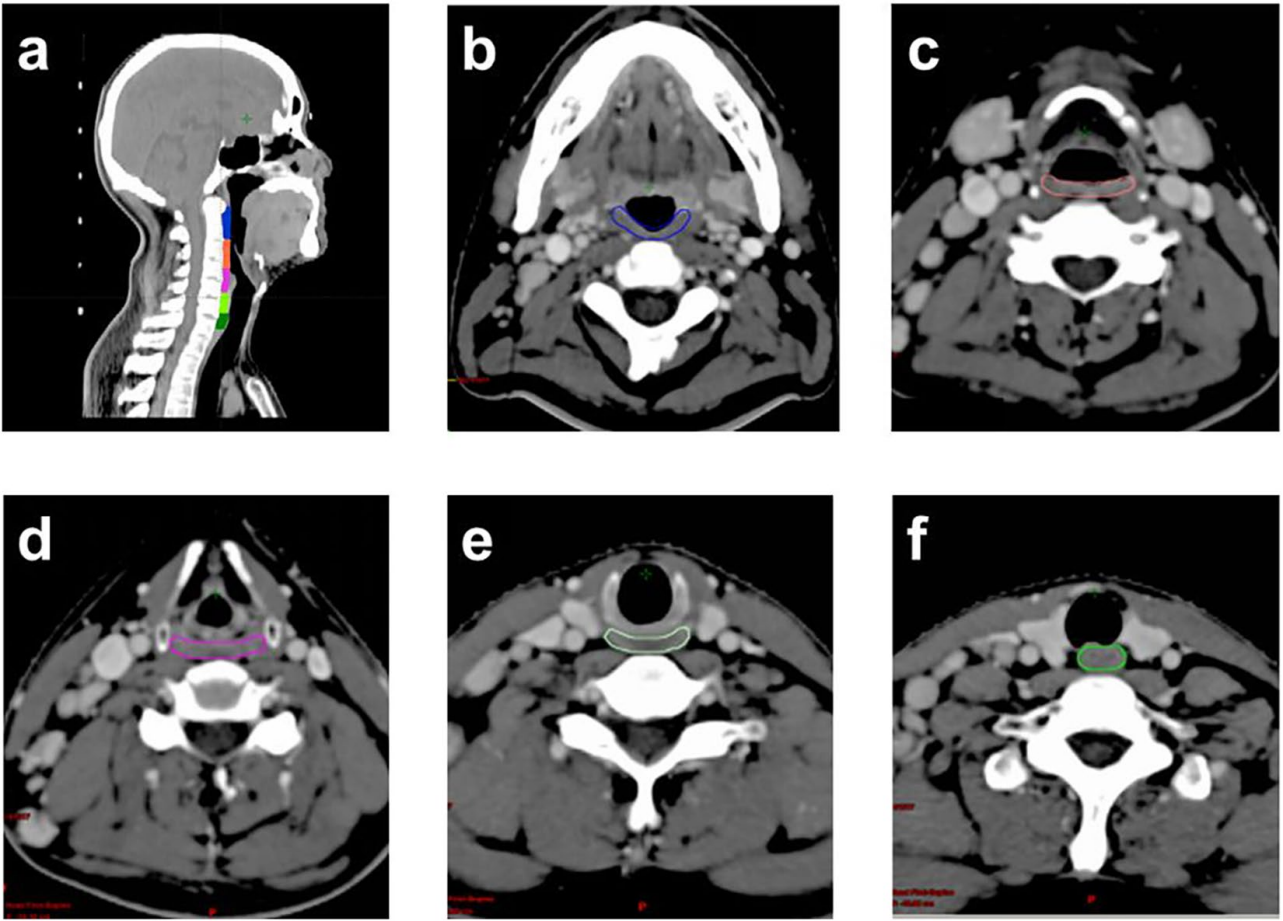
Anatomical delineations of the SWOARs. (**a**) Delineations on sagittal CT-slices. (**b–f**) The contours of the SCM, MCM, ICM, CPM and EIM on axial CT-slices.

Based on published studies^9–12^, the SWOARs comprise of the following five muscles: the superior constrictor muscle (SCM), middle constrictor muscle (MCM) and inferior constrictor muscle (ICM) which are part of the pharyngeal constrictor muscle (PCM), the cricopharyngeus muscle (CPM), and the esophagus inlet muscle (EIM). The SCM, MCM and ICM form the posterior and lateral pharyngeal walls. The SCM extends from the caudal tip of the pterygoid plate to the lower edge of second cervical vertebra, MCM extends from the upper edge of third cervical vertebra to the lower edge of the hyoid bone, and ICM extends from below the lower edge of the hyoid bone to the lower edge of the arytenoid cartilage. CPM extended from below the lower edge of the arytenoid cartilage to the lower edge of the cricoid cartilage, and EIM consisted of the 1 cm of the muscular compartment of the esophagus inlet. Anatomical borders of each SWOAR are indicated in Table 1, and further delineated in Fig. 1.

**Table 1:** Anatomic borders of the SWOARs.

SWOARs	Cranial	Caudal	Anterior	Posterior	Lateral
SCM	Caudal tip of the pterygoid plate	Lower edge of second cervical vertebra	Pterygoid plate or the base of tongue	Prevertebral muscle	Medial pterygoid muscle
MCM	Upper edge of third cervical vertebra	Lower edge of hyoid bone	Base of tongue or the hyoid bone	Prevertebral muscle	Greater horn of the hyoid bone
ICM	Below the lower edge of hyoid bone	Lower edge of arytenoid cartilage	Soft tissue of larynx	Prevertebral muscle	Superior horn of thyroid cartilage
CPM	Below the lower edge of arytenoid cartilage	Lower edge of cricoid cartilage	Posterior margin of the cricoid cartilage	Prevertebral muscle	Thyroid cartilage, soft tissue or thyroid gland
EIM	Below the lower edge of cricoid cartilage	1 cm caudal to esophagus inlet	Posterior margin of the trachea	Prevertebral muscle	Soft tissue or the thyroid gland

Abbreviations: SCM: the superior constrictor muscle, MCM: the middle constrictor muscle, ICM: the inferior constrictor muscle, CPM: cricopharyngeus muscle, EIM: esophagus inlet muscle, cm = centimeter.

### Assessment of dysphagia

All patients were followed-up to assess late dysphagia using the RTOG/EORTC scale^13^. Patient-reported clinical swallowing function was also assessed by the M.D. Anderson dysphagia inventory (MDADI) scoring, which consists of 20 questions with global, emotional, functional and physical subscales. In the MDADI questionnaire, a higher score is equated to a better quality of life and less swallowing trouble^14^.

### Statistical analyses

Age, duration of follow up and dosimetric variables (D_mean_ and V_n_) were analyzed. Data conforming to normal distribution were compared using t-test, and data with non-normal distribution using the Wilcoxon Rank-Sum test. The χ^2^ or Fisher’s test were used to compare the grade 0-1 and grade 2 late dysphagia groups.

The clinical and dosimetric variables were assessed in the univariate analysis, and were incorporated into a binary logistic regression model to assess their independent contribution. Prior to the multivariate analysis, a correlation matrix was produced to identify those potential prognostic factors with high correlations, in particular between the DVH parameters. Based on Pearson correlation coefficients P ≥ 0.70 or variance inflation factor (VIF) ≥ 10 between the candidate prognostic factors, only one variable was selected and incorporated into the model to avoid multicollinearity, which may have negatively affected the generality of the model. The dosimetric variables that resulted from the independent factors of multivariate analysis and their corresponding significant DVH parameters in the univariate analysis were used for receiver operating characteristic (ROC) curve analysis, in order to calculate their threshold values of late dysphagia and assess their diagnostic capability. The dosimetric variables, which showed a correlation in the univariate analysis, were subjected to principal component analysis (PCA). Pearson correlation coefficients (r) were used to analyze the association between MDADI results and all demographic parameters, dosimetric variables and the degree of dysphagia (RTOG/EORTC scale).

Statistical analysis was performed using the STATISTICA 22.0 software. A p-value of ≤ 0.05 was considered statistically significant. The figures were generated using GRAPHPAD 5.0.

## Results

### Patents characteristics and late toxicity

Table 2 summarized the clinical characteristics of 134 patients diagnosed with NPC. The median age of the patients was 44 years (range 18–71 years). During the median follow-up of 34 months (range 25–44 months), 71 patients (53%) were reportedly suffering from late dysphagia. Late dysphagia, according to the RTOG/EORTC scale, was scored as grade (G) 0 in 63 (47%) patients, G1 in 37 (28%) patients and G2 in 34 patients (25%). No cases of G3-4 toxicity were found. We then divided the patients into Group 1 (grade 0–1) and Group 2 (grade 2) based on the severity scores of dysphagia, and found that age and gender were related to the grade level of late dysphagia in the univariate analysis (p < 0.05).

**Table 2:** The relationship between the patients’ clinical characteristics and the RTOG/EORTC degree of late dysphagia.

	Grade 0–1 (n = 100)	Grade 2 (n = 34)	P-Value
Gender (No. of patients)			0.002
Male	82 (82.0%)	19 (55.9%)	
Female	18 (18.0%)	15 (44.1%)	
Age (years) (mean ± SD)	43.3 ± 10.2	48.0 ± 11.0	0.024
Tumor stage (No. of patients)			0.946
T1	6 (6%)	1 (2.9%)	
T2	23 (23%)	7 (20.6%)	
T3	42 (42%)	16 (47.1%)	
T4	29 (29%)	10 (29.4%)	
Nodal stage (No.of patients)			0.417
N0	8 (8%)	1 (2.9%)	
N1	40 (40%)	10 (29.4%)	
N2	47 (47%)	20 (58.8%)	
N3	5 (5%)	3 (8.8%)	
Smoker (No.of patients)			0.371
Yes	41 (41%)	11 (32.4%)	
No	59 (59%)	23 (67.6%)	
Abuse of alcohol (No. of patients)			0.959
Yes	21 (21%)	7 (20.6%)	
No	79 (79%)	35 (79.4%)	
Histology (No. of patients)			0.177
Differentiated non keratinized carcinoma	26 (26%)	5 (14.7%)	
Undifferentiated non keratinized carcinoma	74 (74%)	29 (85.3%)	
Time of follow up(months) (mean ± SD)	34.5 ± 4.5	34.8 ± 4.2	0.730
Neoadjuvant chemotherapy			
Yes	35 (35%)	13 (31.2%)	0.734
No	65 (65%)	21 (61.8%)	
Cisplatin based concurrent chemotherapy			
Yes	86 (86%)	22 (64.7%)	0.007
No	14 (14%)	12 (35.3%)	

### Association between the dosimetric parameters of SWOARs and late dysphagia

**Figure 2:**
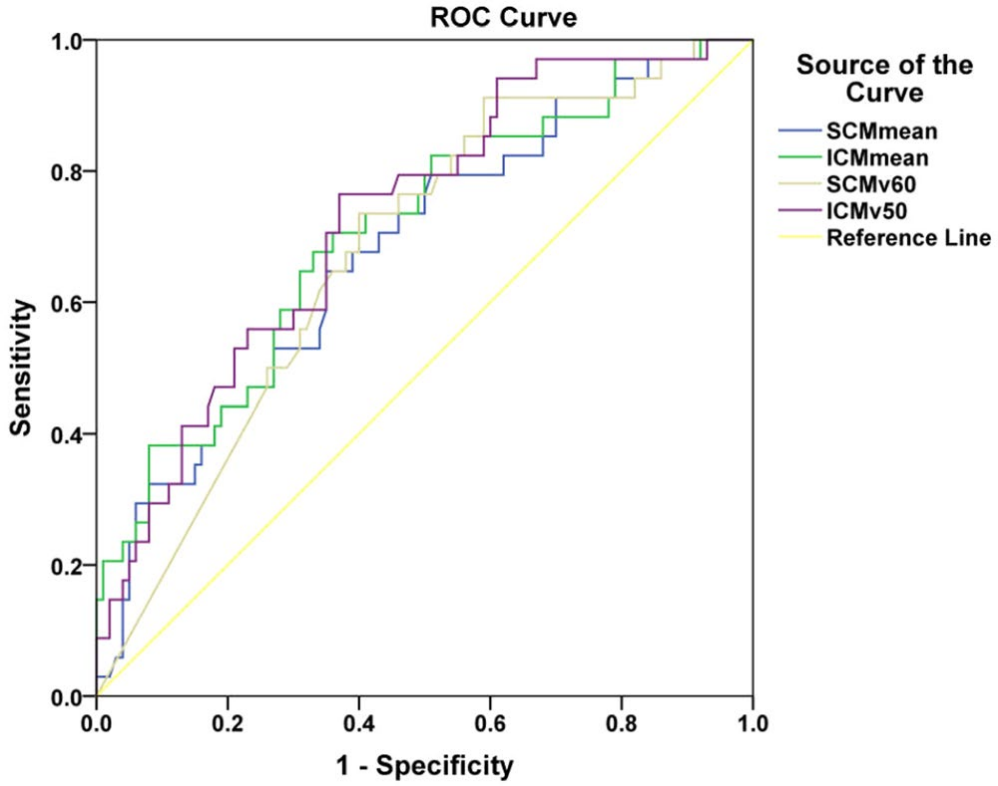
ROC curve of the dosimetric parameters for grade 2 late dysphagia.

The correlation between D_mean_, V_50_, and V_60_ to the affected constrictor muscles and the severity of the late dysphagia is shown in Table 3. The respective D_mean_ to the PCM, SCM, MCM and ICM were each associated with G2 late dysphagia (p ≤ 0.001). Similarly, the respective V_60_ of the SCM and MCM, and V_50_ of the MCM and ICM were correlated with G2 late dysphagia (p ≤ 0.01). Multivariate analysis by forward elimination of insignificant explanatory variables was performed to adjust for various factors. Due to the high correlation between the D_mean_ and the V_50_/V_60_ of the constrictor muscles (Pearson coefficient > 0.8, p < 0.001), we used the D_means_ to SCM and ICM for multivariate analysis, with age and gender as co-variants. Multivariate analysis showed that age (OR 1.050, 95%CI 1.005–1.098, p = 0.031), D_mean_ to SCM (OR 1.170, 95%CI 1.018–1.344, p = 0.027) and D_mean_ to ICM (OR 1.251, 95%CI 1.074–1.457, p = 0.004) were the independent predictors (Table 4). Finally, we evaluated the dose tolerance of the grade 2 late dysphagia using ROC curves in terms of the above significant independent variables. Due to the strong correlation between the V_50_/V_60_ and D_mean_, V_50_ of ICM and V_60_ of SCM were also evaluated using ROC curve analysis. The significant dosimetric parameters and cut-off points of the ROC curve analysis are shown in Table 5. D_mean_ to SCM ≥ 67 Gy, D_mean_ to ICM ≥ 47 Gy, V_60_ of SCM ≥ 95% and V_50_ of ICM ≥ 23% were the threshold values of grade 2 late dysphagia (Supplementary Figures 1–4). The areas under the ROC curves, as showed in Fig. 2, were 0.681 for D_mean_ to SCM (sensitivity 0.647, specificity 0.65, p = 0.002) and 0.71 for D_mean_ to ICM (sensitivity 0.676, specificity 0.67, p < 0.001). In the same way, the areas under the ROC curves were 0.677 for V_60_ of SCM (sensitivity 0.735, specificity 0.6, p = 0.002) and 0.726 for V_50_ of ICM (sensitivity 0.765, specificity 0.63, p < 0.001). In the principal component analysis, KMO and Bartlett tests showed correlations between the included variables (p < 0.001). The scree plot suggested three principle components as the optimal number which could explain 75.3% of the variation. Subsequently, a rotating element matrix was used to identify the dosimetric variables that had different attributes (Table 6). D_mean_ to ICM, V_50_ of ICM and V_60_ of ICM had the most distinguishable contributions to the first principal component. Furthermore, the reliability and validity were examined by MDADI as showed in Table 7. The CPM and EIM were non-significant factors in relation to the grade 2 late dysphagia.

**Table 3:** Relationship between the dosimetric variables of the SWOARs and the degree of late dysphagia.

SWOARs	Grade 0–1	Grade 2	p value
PCM D mean	57.1 ± 3.1	60.3 ± 3.7	** <0.001**
SCM			
D mean	65.8 ± 4.9	68.7 ± 3.8	**0.001**
V_50_	100(100,100)	100(100, 100)	0.281
V_60_	90.6(76.0,100)	99.4(91.9,100.0)	**0.002**
MCM			
D mean	55.9 ± 4.3	58.8 ± 5.2	**0.001**
V_50_	81.7 (68.4,93.6)	95.6(84.7,100.0)	**0.001**
V_60_	28.2 (6.0, 46.2)	39.2 (19.6,65.8)	**0.009**
ICM			
D mean	46.1 ± 3.4	49.5 ± 5.1	**0.001**
V_50_	16.0 (6.2,29.6)	31.9 (21.9,59.8)	**<0.001**
V_60_	0.9 (0.0,3.8)	1.6(0.0,5.1)	0.098
CPM			
D mean	45.2 ± 4.2	45.2 ± 8.7	0.990
V_50_	8.6 (1.2,28.7)	12 (8.2,42.0)	0.108
V_60_	0 (0,0)	0 (0,0)	0.121
EIM			
D mean	42.8 (40.1,45.7)	44.4 (40.5,46.9)	0.223
V_50_	0 (0.0,6.8)	0.6 (0.0,13.5)	0.210
V_60_	0 (0,0)	0 (0,0)	0.755

D, dose; V_50,60,_ partial volume receiving specified dose of 50 Gy; 60 Gy; SCM, superior constrictor muscle; MCM, middle constrictor muscle; ICM, inferior constrictor muscle; CPM, cricopharyngeus muscle; EIM, esophageal inlet muscle.

**Table 4:** Results of binary logistic regression for grade 0–1 and grade 2 late dysphagia patients.

	OR	95%CI	p value
Gender	0.437	0.160–1.197	0.107
Age	1.050	1.005–1.098	0.031
SCM Dmean	1.170	1.018–1.344	0.027
MCM Dmean	1.006	0.875–1.157	0.936
ICM Dmean	1.251	1.074–1.457	0.004

**Table 5:** ROC curve of dosmetric parameters for grade 2 late dysphagia.

	Area	p value	95% CI	Threshold	Sensitivity	Specificity
SCM D_mean_	0.681	0.002	0.577–0.784	67 Gy	0.647	0.65
ICM D_mean_	0.71	<0.001	0.608–0.812	47 Gy	0.676	0.67
SCM V_60_	0.677	0.002	0.578–0.776	95%	0.735	0.60
ICM V_50_	0.726	<0.001	0.632–0.821	23%	0.765	0.63

**Table 6:** Rotating element matrix of the principle component analysis.

	Principle components		
	1	2	3
SCM D_mean_	0.027	0.837	0.162
SCM V_60_	−0.015	0.868	0.117
SCM V_50_	0.182	0.123	0.822
MCM D_mean_	0.562	0.674	−0.348
MCM V_60_	0.439	0.722	−0.161
MCM V_50_	0.483	0.540	−0.480
ICM D_mean_	**0.948**	0.079	−0.013
ICM V_60_	**0.654**	0.111	0.174
ICM V_50_	**0.885**	0.113	0.019

**Table 7:** Pearson coefficient of MDADI and variable quantities.

	Pearson coefficient	p value
Age	−0.252	0.003
SCM D_mean_	−0.177	0.041
SCM V_60_	−0.236	0.006
ICM D_mean_	−0.292	0.001
ICM V_50_	−0.278	0.001

## Discussion

Although IMRT with concomitant chemotherapy for NPC has a high rate of local control, radiation-induced late dysphagia is usually debilitating to patients. Furthermore, late dysphagia in NPC patients treated with IMRT is poorly understood. It is essential to know the dose tolerance for dysphagia in order to predict the safety of treatment plans. The aim of this retrospective study was to improve the understanding of late dysphagia and thus optimize IMRT for NPC.

The effect of the radiation dose and volume on the healthy tissues is a major concern in radiotherapy. Patterson *et al*. evaluated the swallowing function of 18 NPC patients using a patient-reported oral function score with fiberoptic endoscopic examination; 5 patients suffered from moderate late dysphagia but no severe cases were observed^4^. Another study by Xu *et al*. showed that 50% of NPC patients treated with IMRT had swallowing dysfunction^15^. Our results, which are consistent with the previous studies, showed that 53% of the patients experienced late dysphagia and grade 2 late dysphagia occurred in 25% of the patients. Therefore, treatment-related late toxicity may worsen patients’ quality of life.

Some recent studies have assessed the relationship between the dose-volume parameters of swallowing structures and late dysphagia in patients undergoing treatment for head and neck tumors^9,16,17^. Deantonio *et al*. reported that >50 Gy D_mean_ to SCM and MCM correlated with grade 2–3 late dysphagia, and D_mean_ to MCM is the only significant predictor of late grade dysphagia^18^. In the prospective study of Feng *et al*., patients with adequate aspiration received >60 Gy D_mean_ to PCM^19^. Furthermore, studies have also analyzed the incidence of late dysphagia relative to tumor location. The primary tumor site in the larynx, hypopharynx and posterior pharyngeal wall was associated with long-term dysphagia^20–22^. However, very few studies have been conducted on late dysphagia in NPC patients who received IMRT, and did not reveal any significant association between late dysphagia and dose-volume effect. A study found that the SCM, MCM and ICM easily tolerated radiation doses in patients with a small tumor volume, but had a lower tolerance in case of larger tumors^23^. Our results demonstrated that the doses delivered to the SCM and ICM were independent factors predicting G2 late dysphagia. Consequently, we found that the D_mean_ to SCM ≥ 67 Gy, V_60_ of SCM ≥ 95%, D_mean_ to ICM ≥ 47 Gy and V_50_ of ICM ≥ 23% were the threshold doses of G2 late dysphagia in NPC patients treated with IMRT. A study evaluated the anatomical changes in the PCM after chemo-radiotherapy of head and neck cancer and their dose-effect relationships using MRI^7^. The MRI signals and the muscle thickness changed significantly as the dose increased, suggesting that the underlying causes of SWOARs dysfunction are inflammation and edema. On the other hand, Truong *et al*. reported that late dysphagia was related to radiation-induced free radical damage to the SWOARs and subsequent development of PCM fibrosis, with stricture formation and loss of muscle pliability^24^. They also found that oxidative stress and microvascular injury to the endothelium correlated with progressive changes in the blood. Therefore, tumor location and radiation dose to the SWOARs are significant factors in late dysphagia. Levendag *et al*. found significant relationships between the D_mean_ > 50 Gy to the SCM and MCM and severe dysphagia complaints^10^. Christianen *et al*. also showed that late dysphagia correlated with D_mean_ > 60 Gy to SCM or MCM^25^. Based on the results of these two studies, the SCM and MCM likely have a major role in late dysphagia, while ICM may not influence swallowing symptoms. In contrast, Dirix *et al*. indicated that a D_mean_ > 50 Gy to the MCM and ICM significantly correlated with late dysphagia. Nevertheless, our results showed that the dose delivered to ICM also had a crucial impact on late dysphagia, as did between the dose and SCM, whereas the dose delivered to MCM had no impact.

IMRT is widely used for patients with NPC, and delivers a high radiation dose to tumors while maintaining a safe dose for normal tissues surrounding the tumor. This technique also exhibits excellent tumor coverage. Compared to three-dimensional conformational radiation therapy, IMRT in head and neck reduces adverse effects such as dysphagia and thus improves quality of life (QOL)^26,27^. Currently, despite expert recommendations to spare a portion of the SWOARs in order to reduce dysphagia, the dose constraint to PCM is unclear^26,28^. Studies have shown different results, possibly due to methodological differences and the ambiguous contouring of the SWOARs. Limiting the radiation dose to the crucial SWOARs is expected to decrease the incidence and severity of radiation-induced dysphagia with IMRT. A study used the new technique of swallowing sparing IMRT (SW-IMRT), and reduced the doses to the SWOARs based on the following criteria listed in order of priority: (1) minimizing the mean dose to the SCM, (2) minimizing the mean dose to the MCM, (3) minimizing the mean dose to the supraglottic larynx, and (4) minimizing the proportion of the EIM receiving ≥60 Gy (EIM V_60_). Compared to the standard IMRT (ST-IMRT), SW-IMRT reduced the mean dose to the various SWOARs, along with a 9% mean reduction (3%-20%) in predicted physician-rated RTOG/EORTC grade 2–4 swallowing dysfunction^27^.

The results of our study must be viewed cautiously because of several limitations. Due to the retrospective approach, we used the previously described dose tolerance in clinical practice in a prospective manner till they were validated. The second limitation is regarding the method of delineation of the swallowing structures that, although performed by an experienced radiation oncologist, are not fully standardized and could thus result in a bias. Other limitations are the small sample size, lack of anatomical examinations such as endoscopy, MRI, and barium meal test.

## Conclusion

We found a significant relationship between late dysphagia and the radiation dose delivered to superior and inferior pharyngeal constrictor muscles during radiotherapy of NPC. The D_mean_ to SCM ≥ 67 Gy, V_60_ of SCM ≥ 95%, D_mean_ to ICM ≥ 47 Gy, and V_50_ of ICM ≥ 23% correlated with grade 2 late dysphagia. The optimal modality to assess late dysphagia and the most appropriate dose limits of constrictor muscles are still open issues and should be further investigated. A prospective study is needed to validate the findings of the present study, and to determine whether the dosimetric benefits of this treatment strategy translate into better clinical outcomes.

## Electronic supplementary material

Supplementary Figures
