# Supplementary material for: Radiation-induced late dysphagia after intensity-modulated radiotherapy in nasopharyngeal carcinoma patients: a dose-volume effect analysis
Source: Sci Rep. 2018 Nov 6;8:16396. doi: 10.1038/s41598-018-34803-y (PMC6219576; doi:10.1038/s41598-018-34803-y)
Supplement: Supplementary file 1 — Supplementary Figures [file 41598_2018_34803_MOESM1_ESM.pdf]

Radiation-induced late dysphagia after intensity-modulated radiotherapy in nasopharyngeal carcinoma patients: a dose-volume effect analysis

Li Jiang[1#], Chenhui Huang[2#], Yixiu Gan[1#], Tong Wu[1], Xiaobi Tang[1], Yiru Wang[1], Rensheng Wang[1*], Yong Zhang[1*]

1 Department of Radiation Oncology, The First Affiliated Hospital of Guangxi Medical University, Radiation Oncology Clinical Medical Research Center of Guangxi, Nanning, Guangxi, China

2 Guangxi clinical research center for digital medicine and 3D printing, Guigang City People's Hospital, Guigang, Guangxi, China

Supplementary Figures

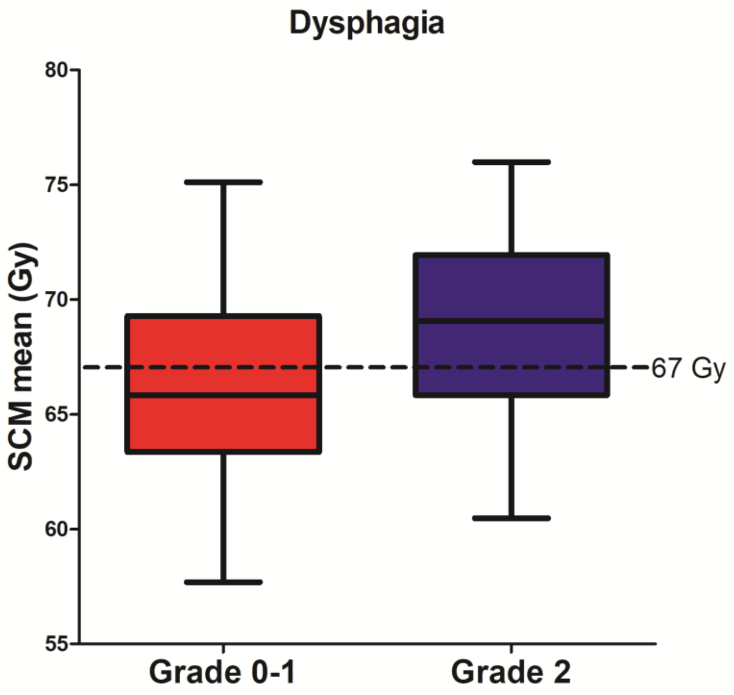

Supplementary Figure 1. Threshold value of the D_{mean} to the SCM for predicting grade 2 late dysphagia.

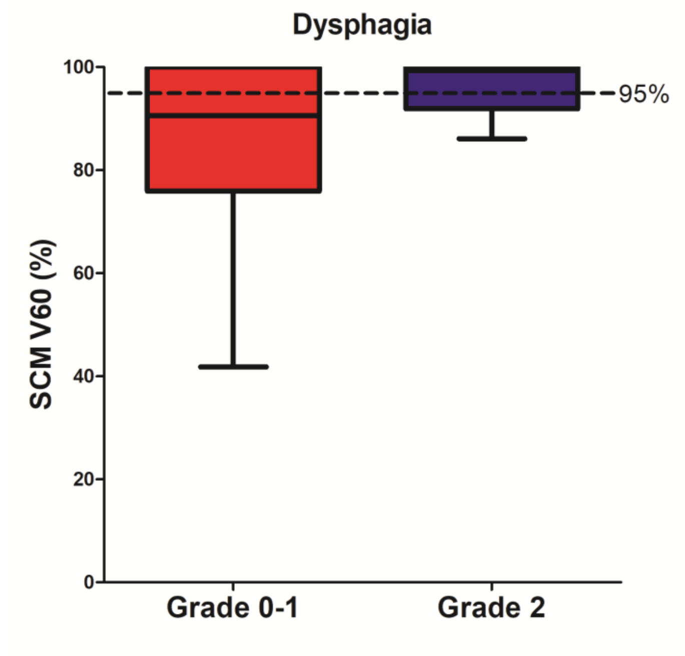

Supplementary Figure 2. Threshold value of the V_{60} of the SCM for predicting grade 2 late dysphagia

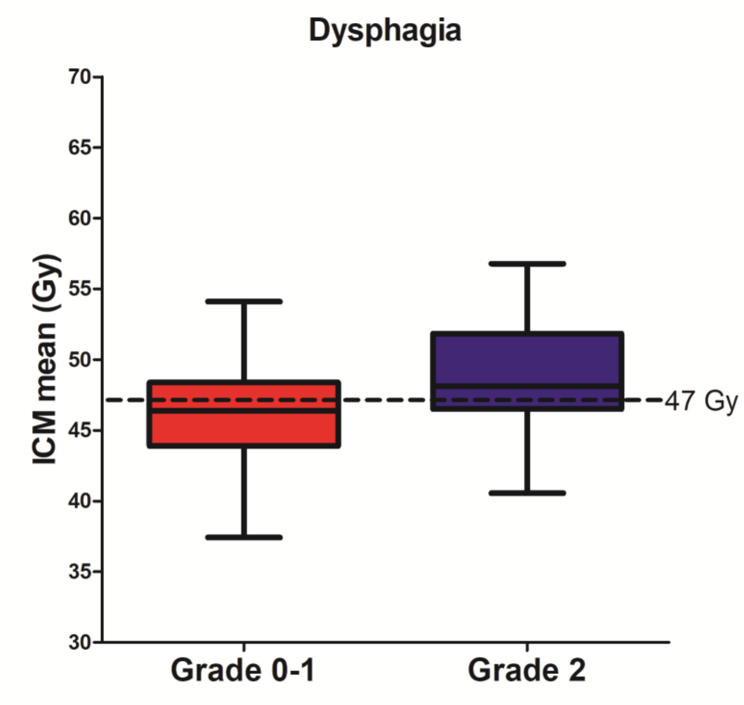

Supplementary Figure 3. Threshold value of the D_{mean} to the ICM for predicting grade 2 late dysphagia.

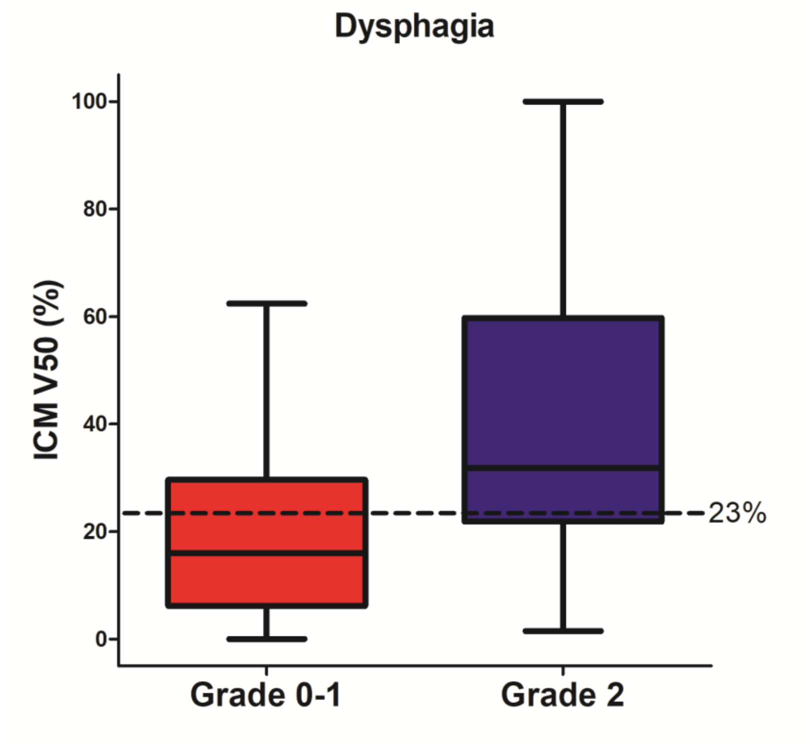

Supplementary Figure 4. Threshold value of the V_{50} of the ICM for predicting grade 2 late dysphagia
